# Melanin is an essential component for the integrity of the cell wall of *Aspergillus fumigatus *conidia

**DOI:** 10.1186/1471-2180-9-177

**Published:** 2009-08-24

**Authors:** Marc Pihet, Patrick Vandeputte, Guy Tronchin, Gilles Renier, Patrick Saulnier, Sonia Georgeault, Romain Mallet, Dominique Chabasse, Françoise Symoens, Jean-Philippe Bouchara

**Affiliations:** 1 Laboratoire de Parasitologie-Mycologie, Centre Hospitalier Universitaire d'Angers, France; 2 Groupe d'Etude des Interactions Hôte-Pathogène, UPRES-EA 3142, Université d'Angers, France; 3 INSERM U646, Université d'Angers, France; 4 Service Commun d'Imageries et Analyses microscopiques, Université d'Angers, France; 5 Scientific Institute of Public Health, Brussels, Belgium

## Abstract

**Background:**

*Aspergillus fumigatus *is the most common agent of invasive aspergillosis, a feared complication in severely immunocompromised patients. Despite the recent commercialisation of new antifungal drugs, the prognosis for this infection remains uncertain. Thus, there is a real need to discover new targets for therapy. Particular attention has been paid to the biochemical composition and organisation of the fungal cell wall, because it mediates the host-fungus interplay. Conidia, which are responsible for infections, have melanin as one of the cell wall components. Melanin has been established as an important virulence factor, protecting the fungus against the host's immune defences. We suggested that it might also have an indirect role in virulence, because it is required for correct assembly of the cell wall layers of the conidia.

**Results:**

We used three *A. fumigatus *isolates which grew as white or brown powdery colonies, to demonstrate the role of melanin. Firstly, sequencing the genes responsible for biosynthesis of melanin (*ALB1*, *AYG1*, *ARP1*, *ARP2*, *ABR1 *and *ABR2*) showed point mutations (missense mutation, deletion or insertion) in the *ALB1 *gene for pigmentless isolates or in *ARP2 *for the brownish isolate. The isolates were then shown by scanning electron microscopy to produce numerous, typical conidial heads, except that the conidia were smooth-walled, as previously observed for laboratory mutants with mutations in the *PKSP/ALB1 *gene. Flow cytometry showed an increase in the fibronectin binding capacity of conidia from mutant isolates, together with a marked decrease in the binding of laminin to the conidial surface. A marked decrease in the electronegative charge of the conidia and cell surface hydrophobicity was also seen by microelectrophoresis and two-phase partitioning, respectively. Ultrastructural studies of mutant isolates detected considerable changes in the organisation of the conidial wall, with the loss of the outermost electron dense layer responsible for the ornamentations seen on the conidial surface in wild-type strains. Finally, analysis of the conidial surface of mutant isolates by atomic force microscopy demonstrated the absence of the outer cell wall rodlet layer which is composed of hydrophobins.

**Conclusion:**

These results suggest that, in addition to a protective role against the host's immune defences, melanin is also a structural component of the conidial wall that is required for correct assembly of the cell wall layers and the expression at the conidial surface of adhesins and other virulence factors.

## Background

*Aspergillus fumigatus*, the most common agent of human and animal aspergillosis, is an opportunistic mould responsible for various infections in receptive hosts, ranging from colonisation of the airways in patients with cystic fibrosis to severe and often fatal disseminated infections in immunocompromised patients [1].

**Figure 1:**
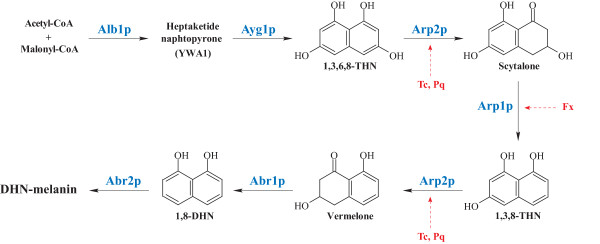
**Biosynthetic pathway of melanin in *A. fumigatus***. White mutants obtained by Brakhage [5] and Kwon-Chung [4] had mutations in the *ALB1 *(also called *PKSP*) gene. Steps inhibited by commercialised DHN-melanin inhibitors are localized (Tc, tricyclazole; Pq, pyroquilon; Fx, fenoxanil). 1,3,6,8-THN, 1,3,6,8-tetrahydroxynaphthalene; 1,3,8-THN, 1,3,6,8-trihydroxynaphthalene; DHN, dihydroxynaphthalene (adapted from Tsai *et al*. [35]).

Elucidation of the pathogenesis of these infections has been the subject of many scientific investigations over the last few years [2,3]. It has been suggested that numerous fungal components play a role in pathogenesis, including adhesins and hydrophobins, proteases or phospholipases, catalases and superoxide dismutases or non ribosomal peptide synthases involved in the synthesis of hydroxamate-type siderophores (for a review, see reference [1]). In addition, several virulence factors have been discovered such as gliotoxin, components involved in iron and zinc acquisition or in various signalling pathways, and melanin [1]. The latter is synthesized through the dihydroxynaphtalene (DHN)-melanin pathway (Figure 1) in *A. fumigatus*. Its biosynthesis involves 6 genes, organized in a cluster, which are expressed during conidiation. This complex metabolic pathway starts with acetyl-CoA and malonyl-CoA which are converted by the products of the genes *PKSP *(also called *ALB1*) and *AYG1 *into 1,3,6,8 tetrahydroxynaphtalene (THN). Then, by successive steps of reduction (catalyzed by the product of the gene *ARP2*) and dehydration (catalysed by the scytalone dehydratase and the vermelone dehydratase, encoded by the genes *ARP1 *and *ABR1*, respectively), 1,3,6,8-THN is in turn converted to 1,8-DHN, which is finally polymerised by a fungal laccase encoded by the *ABR2 *gene. Strains with mutations in the *PKSP*/*ALB1 *gene were obtained by exposure to UV or by gene disruption and were shown to be less virulent than their parent wild-type strains in murine models of disseminated aspergillosis [4,5]. *In vitro *experiments showed that melanin protects the conidia from phagocytosis and increases their resistance to reactive oxygen species produced by phagocytic cells [4,6]. However, deletion of the *ABR2 *gene in a wild-type strain did not reduce virulence in an intranasal mouse infection model [7].

**Figure 2:**
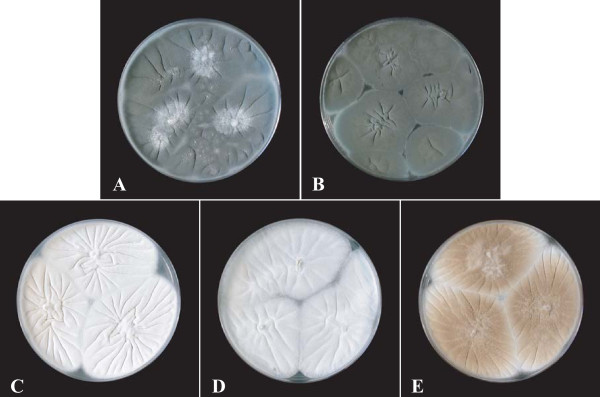
**5-day-old cultures of the different strains or isolates studied on YPDA plates**. Reference strains CBS 113.26 (**A**) and IHEM 18963 (**B**) produce typical dark-blue green powdery colonies, whereas mutant isolates IHEM 2508 (**C**), IHEM 9860 (**D**) produce white powdery colonies and IHEM 15998 (**E**), brown powdery colonies.

Adherence of microorganisms to the host tissues is considered a crucial step in the initiation of infection. Previous studies on *A. fumigatus *by our group [8,9] and others [10,11] suggested that specific interactions involving the recognition of the extra-cellular matrix (ECM) component proteins, laminin and fibronectin, could mediate adherence. Immunofluorescence studies and scanning or transmission electron microscopy (SEM or TEM) also suggested that fungal adhesins for the ECM proteins are located on the ornamentations of the cell wall of resting conidia, the agents of infection. Therefore, as it had been shown by SEM that laboratory strains with mutations in the *ALB1/PKS *gene produce smooth-walled conidia, we predicted that melanin also plays an indirect role in pathogenesis, allowing correct assembly of the cell wall layers of resting conidia. In this study, three pigmentless or brownish isolates of clinical or environmental origin, from the BCCM/IHEM Collection (Scientific Institute of Public Health, Brussels, Belgium), were investigated and compared to two reference strains (Figure 2 and Table 1). After characterisation of the genetic defect of the three mutant isolates and visualisation of the conidial surface by SEM, the capacity of their conidia to bind the ECM components laminin and fibronectin was quantified and the physical properties of the conidial surface were investigated. Finally, an ultrastructural study of the conidial wall was performed, and the surface of the conidia was analysed by atomic force microscopy (AFM).

**Table 1:** Origin of the mutant isolates studied

IHEM number	Colonies on YPDA	Year of isolation	Origin of sample	Country of isolation
**2508**	White powdery	1985	Hospital environment	Belgium
**9860**	White powdery	1975	Cultivated soil	India
**15998**	Brown powdery	1999	Human sputum (patient with cystic fibrosis)	France

## Results

### Susceptibility to dihydroxy-naphtalene (DHN)-melanin inhibitors and characterisation of the genetic defect

**Figure 3:**
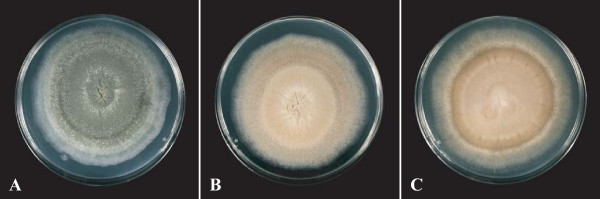
**Effects of pyroquilon on colony colour of *A. fumigatus *grown on Czapek medium**. The reference strain CBS 113.26 was grown on Czapek agar, supplemented (**B**) or not (**A**) with 20 μg/mL of pyroquilon. The colour of the colonies obtained in the presence of this inhibitor of the melanin biosynthesis pathway is similar to that of colonies of the brownish isolate IHEM 15998 grown on Czapek medium (**C**).

To identify which steps of the melanin biosynthesis pathway were affected in mutant isolates, the effect of specific DHN-melanin inhibitors was analysed based on colony colour and radial growth on culture media supplemented with tricyclazole, pyroquilon or fenoxanil. Tricyclazole and pyroquilon inhibit hydroxynaphtalene reductase encoded by the *ARP2 *gene, while fenoxanil interferes with scytalone dehydratase encoded by the *ARP1 *gene (Figure 1). On Czapek medium supplemented with 20 μg/mL of tricyclazole, pyroquilon or fenoxanil, *A. fumigatus *CBS 113.26 and IHEM 18963 developed powdery colonies with pigmentation similar to that of colonies of the brownish isolate IHEM 15998 (Figure 3). The inhibitors had no effect on pigmentless or brownish isolates. The colour of the colonies of these mutant isolates was not affected, nor was their diameter significantly modified in most cases (Table 2).

**Table 2:** Growth on Czapek medium supplemented with inhibitors of melanin biosynthesis

Strain or isolate number	Control	Tricyclazole	Pyroquilon	Fenoxanil
**Reference strains**				
**CBS 113.26**	31.7 ± 1.52	30 ± 4.36	29.3 ± 2.08	32.3 ± 0.58
**IHEM 18963**	32 ± 2	31.7 ± 1.15	28 ± 1*	31.2 ± 0.28
				
**Mutant isolates**				
**IHEM 2508**	33.7 ± 0.58	32 ± 2	31 ± 1*	33.3 ± 1.15
**IHEM 9860**	31.7 ± 1.15	30.7 ± 1.53	34 ± 1.73	25.3 ± 1.53*
**IHEM 15998**	35.7 ± 0.58	34 ± 1.73	35 ± 2.64	27.7 ± 0.58*

Experiments were performed in triplicate and results are expressed as mean diameter (mm) of the colonies (± standard deviation) after 72 hours of incubation at 37°C. *indicates statistically significant difference between control and inhibitor of melanin biosynthesis (unpaired Student's *t*-test; *P *< 0.05).

The different genes involved in melanin biosynthesis were then amplified by PCR and sequenced, for each strain or isolate. The data obtained were compared with available sequences in the GenBank database (National Institute of Health). Point mutations in *ALB1*, encoding a pentaketide synthase which is involved in the early steps of this metabolic pathway, were identified for pigmentless isolates IHEM 2508 and 9860 (Table 3). More precisely, a nonsense mutation was identified for isolate IHEM 2508, which caused truncation of the enzyme by173 amino acid residues at its C-terminus, leading to the loss of the thioesterase/claisen cyclase (TE/CLC) domain in particular. A deletion was detected for IHEM 9860, leading to a shift in the reading frame from the amino acid at position 1678, and thus to the loss of an acyl carrier protein (ACP) domain and the TE/CLC domain. The metabolic pathway was blocked at a later step for the brownish isolate IHEM 15998. Sequencing of the different genes showed an insertion in the *ARP2 *gene, which encodes a hydroxynaphthalene reductase (Table 3). This mutation led to a shift in the reading frame after the amino acid at position 140, and consequently to the loss of the dehydrogenase/reductase domain. The missense mutation (C1391G) found in *ABR2 *for IHEM 9860 led to the replacement of a glutamine (Gln) by a glutamic acid (Glu) at position 217. The effect of this mutation on the protein function is not clear.

**Table 3:** Mutations detected in the genes involved in melanin biosynthesis for *A. fumigatus *isolates IHEM 2508, 9860 and 15998

Isolate	Point mutations in genes^a^					
	
	*ALB1*	*AYG1*	*ARP2*	*ARP1*	*ABR1*	*ABR2*
**IHEM 2508**	*(FJ406465)*	*(FJ406471)*	*(FJ406477)*	*(FJ406483)*	*(FJ406489)*	*(FJ167495)*
	G1203A^b^	C1017A^b^	G843T	*-*	A677C^b^	A582G^b^
	A4636T^b^		T1053C^b^			
	T5639C^b^					
	**C6739T**					
						
**IHEM 9860**	*(FJ406466)*	*(FJ406472)*	*(FJ406478)*	*(FJ406484)*	*(FJ406490)*	*(FJ167496)*
	C720T	C1017A^b^	T1053C^b^	*-*	A677C^b^	A582G^b^
	G1203A^b^				T594A	
	A4636T^b^				C1391G	
	T5639C^b^					
	**G5854X**					
	G5904A					
						
**IHEM 15998**	*(FJ406468)*	*(FJ406474)*	*(FJ406480)*	*(FJ406486)*	*(FJ406492)*	*(FJ167498)*
	G1203A^b^	C1017A^b^	**X751G**	-	A677C^b^	A582G^b^
	A4636T^b^		G843T			
	T5639C^b^		T1053C^b^			

^a ^Mutations are described as follow: first letter corresponds to the nucleotide present in the GenBank database sequence for the corresponding gene (accession numbers; AF025541, AF116902, AF099736, AFU95042, AF116901, AF104823 for *ALB1*, *AYG1*,*ARP2*, *ARP1*, *ABR1 *and *ABR2*, respectively), the number represents the relative position from the start of the reference sequence, and the second letter represents the nucleotide found in the gene sequence for isolates IHEM 2508, 9860 or 15998. The letter X placed after the number indicates a deletion of the corresponding nucleotide, and the same letter placed before the number corresponds to an insertion. The missense mutations found in the different gene sequences are underlined. Nonsense mutations, insertions and deletions are in bold type. Other mutations correspond to silent mutations. Mutations which correspond to polymorphism outside the encoding sequences are not presented here. GenBank accession numbers of the corresponding sequences are in brackets.

^b ^Mutations shared by the three mutant isolates and the two wild-type strains used as controls. All these mutations were silent, corresponding only to polymorphism, except mutations G1203A (replacement of an aspartic acid by an asparagine) and T5639C (replacement of a phenylalanine by a serine) from comparisons to gene sequences available in the Genbank database.

### Evidence for conidiation and visualisation of the conidial surface by scanning electron microscopy

**Figure 4:**
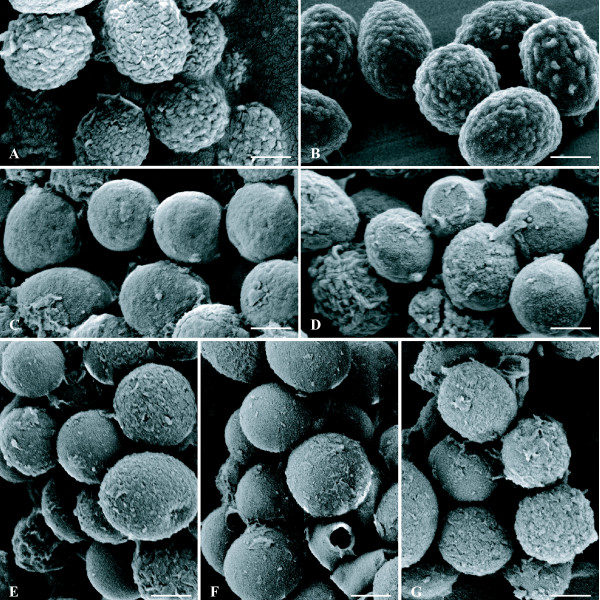
**Visualisation of the conidial surface by scanning electron microscopy**. Conidia from 5-day-old cultures of the reference strains CBS 113.26 (**A **and **C**) and IHEM 18963 (**B **and **D**) cultivated in the presence (**C **and **D**) or not (**A **and **B**) of pyroquilon 20 μg/mL, and of mutant isolates (**E **and **F**: pigmentless isolates IHEM 2508 and 9860; **G**: brownish isolate IHEM 15998) were observed by scanning electron microscopy. Bars correspond to 1 μm.

SEM observation of cultures of mutant isolates on yeast extract - peptone - dextrose - agar (YPDA) plates through dialysis membranes showed typical conidial heads, consistent with the powdery texture of their colonies **(**data not shown). Further examination of the conidia by SEM showed, as expected, a typical echinulate surface for reference strains (CBS 113.26 and IHEM 18963) and smooth-walled conidia for the pigmentless isolates IHEM 2508 and 9860 (Figure 4). SEM also revealed the absence of ornamentations on the conidial surface for the brownish isolate IHEM 15998, as well as for reference strains cultivated in the presence of pyroquilon (Figure 4).

### Flow cytometry analysis of laminin and fibronectin binding

The conidial adhesion to laminin and fibronectin was quantified by flow cytometry on conidia from 5-day-old cultures. Results showed a slight, but significant, increase in specific binding (total binding - non specific binding) of fibronectin at the conidial surface for pigmentless (IHEM 2508 and 9860) and brownish (IHEM 15998) isolates compared to the wild-type strains (CBS113.26 and IHEM 18963), associated with a marked decrease in binding of laminin (Table 4).

**Table 4:** Flow cytometry analysis of the binding of laminin and fibronectin

Strain or isolate number	Control	Laminin binding			Fibronectin binding		
		
		Total	Residual	Specific	Total	Residual	Specific
**Reference strains**							
**CBS 113.26**	20	11442	2054	9388	234	96	138
**IHEM 18963**	37	12652	2792	9860	229	146	83
**Mutant isolates**							
**IHEM 2508**	40	1671	869	802	222	76	146
**IHEM 9860**	63	4606	2465	2141	560	247	313
**IHEM 15998**	35	10785	3574	7211	354	151	203

Results are mean values of the data collected for 10,000 cells. Significance of the difference between two fluorescence frequency distribution histograms was confirmed by statistical analysis using the Kolmogorov-Smirnoff two sample test.

### Evaluation of the physical properties of the conidial surface

The conidial cell surface electrostatic charge was assessed by microelectrophoresis with a Zetasizer and the cell surface hydrophobicity (CSH) was assessed by two-phase partitioning with hexadecane as the hydrocarbon phase or using a two-aqueous phase system. Results showed that the electronegative charge of the conidial surface for mutant isolates was much lower than that of the wild-type strains (Table 5). Likewise, two-phase partitioning showed a decrease in CSH for conidia of pigmentless or brownish isolates. This decreased hydrophobicity is consistent with the increased wettability observed during the preparation of conidial suspensions.

**Table 5:** Physical properties of the conidial surface

Strain or isolate number	Zeta potential (mV)	Water/hexadecane (%)^1^	PEG/dextran^2^
**Reference strains**			
**CBS 113.26**	- 43.8	10	2.37
**IHEM 18963**	- 39.1	11	2.8
**Mutant isolates**			
**IHEM 2508**	- 21.5	2	2.04
**IHEM 9860**	- 26	0.05	1.14
**IHEM 15998**	- 25.6	2.2	1.8

^1 ^Results are expressed as the percentage of conidia that were excluded from the aqueous phase.

^2 ^Results are expressed as the ratio between the absorbance of the upper phase (rich in PEG and hydrophobic) and that of the lower phase (rich in dextran and hydrophilic)

### Ultrastructure of the conidial wall visualised by transmission electron microscopy

**Figure 5:**
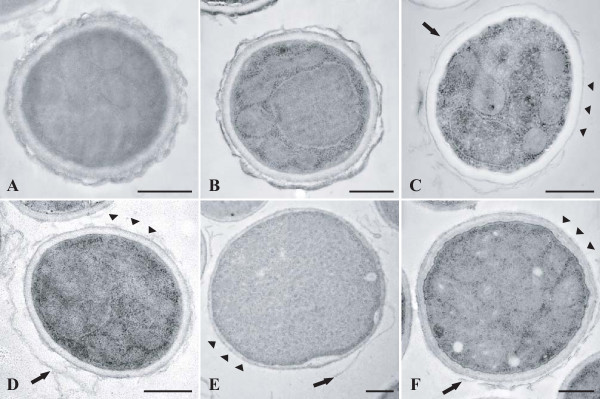
**Ultrastructure of the conidial wall as visualised by transmission electron microscopy**. Conidia from reference strains CBS 113.26 (**A**) and IHEM 18963 (**B **and **C**) cultivated in the presence (**C**) or not (**A **and **B**) of pyroquilon 20 μg/mL, or of mutant isolates (**D **and **E**: pigmentless isolates IHEM 2508 and 9860; **F**: brownish isolate IHEM 15998) were processed for ultrastructural examination of their cell wall. Note the smooth surface of the conidia of reference strains cultivated in the presence (**C**) of pyroquilon and mutant isolates (**D, E, F**) and the lack of the outermost cell wall layer (arrowheads) which sometimes appears free in the surrounding medium (arrows). Bars correspond to 500 nm.

The conidial wall of reference strains was composed of several superimposed layers, with a thick electron transparent inner layer and two thin electron dense outer layers, the outermost layer being responsible for the ornamentations of the cell wall (Figure 5). However, conidia of mutant isolates, as well as those from reference strains cultivated in the presence of pyroquilon, showed a thinner cell wall devoid of the outermost layer which could sometimes be seen free in the surrounding medium.

### Visualisation of the hydrophobic rodlet layer by atomic force microscopy

**Figure 6:**
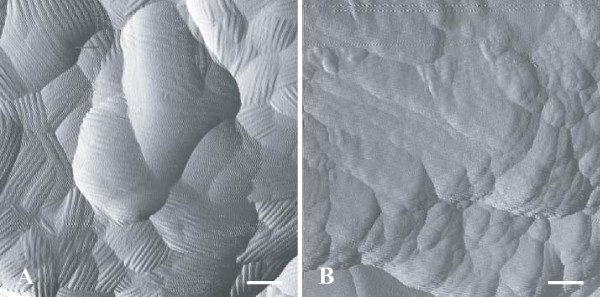
**Images generated by AFM (tapping mode) of the surface of *A. fumigatus *conidia**. Conidia from reference strain IHEM 18963 (**A**) or from brownish isolate IHEM 15998 (**B**) were processed for visualisation of their surface by AFM. Amplitude images show the lack of the hydrophobic rodlet layer at the conidial surface for mutant isolate. Bars correspond to 100 nm.

We also investigated the presence of a hydrophobic rodlet layer on the conidial surface, to provide support for our hypothesis. This protein film is usually composed of about 10-nm thick rodlets of varying length organized into bundles or fascicles, in which individual rodlets lie parallel within a single fascicle. Conidia from 5-day-old cultures of IHEM 18963, IHEM 9860 and IHEM 15998 isolates were therefore examined by AFM. Typical rodlets were detected for the reference strain (IHEM 18963), whereas the rodlet layer seemed to be lacking in conidia of pigmentless (IHEM 9860) or brownish (IHEM 15998) isolates (Figure 6).

## Discussion

Many fungal species produce pigments such as melanin, either from L-3,4-dihydroxyphenylalanine (the DOPA-melanin pathway, which is more frequently encountered in Basidiomycetes) or from 1,8-dihydroxynaphthalene (the DHN-melanin pathway, usually found in Ascomycetes and relative Deuteromycetes) [12]. The genes and enzymes involved in these metabolic pathways have been known for many years, but the two types of melanin were only recently related to virulence in phytopathogenic or human pathogenic fungi [12-14]. For example, DHN-melanin provides the rigidity of appressoria, which allow the fungus to penetrate plant leaves, in *Magnaporthe grisea*, the agent responsible for rice blast [15], and in *Colletotrichum lagenarium*, responsible for cucurbits disease [16]. The role of melanin in virulence is less well defined in human pathogens such as *Cryptococcus neoformans *[17], *Paracoccidioides brasiliensis *[18], *Exophiala dermatitidis *[19] and *Sporothrix schenckii *[20]. It has been demonstrated that this pigment protects the fungal cells especially from reactive oxygen species produced by the host immune defences. Brakhage [5] and Kwon-Chung [4] demonstrated the importance of melanin for *A. fumigatus*. They generated white mutants either by UV mutagenesis, or by targeted mutagenesis. These mutants produced white colonies and had mutations in the *PKSP *(= *ALB1*) gene, encoding a polyketide synthase required for conidial pigmentation. They were less virulent than their parent wild-type strains in murine models of disseminated aspergillosis, probably due to an increased susceptibility of their conidia to phagocytosis and reactive oxygen species. However, virulence in mice was not affected by the disruption of the *ABR2 *gene which is involved in a later step of the melanin pathway [7].

Mutation in the *PKSP *(*ALB1*) gene also led to morphological changes of the conidia. Indeed, SEM showed that these pigmentless mutants produced smooth-walled conidia, whereas the conidia of *A. fumigatus *have typically a rough surface covered with echinulations [5]. The study of mutant isolates of clinical or environmental origin, with defective melanin biosynthesis pathways, suggests that the pigment also plays an indirect role in virulence of *A. fumigatus*.

Sequencing of the genes involved in the melanin biosynthesis pathway showed a genetic defect in the early steps of this pathway for our isolates. This was consistent with the changes in colony colour observed for reference strains grown in the presence of specific DHN-melanin inhibitors. Two distinct mutations in the *ALB1 *gene were detected for IHEM 2508 and 9860 isolates, leading to the production of white powdery colonies; whereas the genetic defect was localised in the *ARP2 *gene for isolate IHEM 15998, producing brown, powdery colonies. As expected, SEM examination of conidial suspensions from our pigmentless isolates showed a smooth surface. However, a lack of ornamentation was also observed on the conidial surface for the brownish isolate, as well as in reference strains cultivated in the presence of pyroquilon, an inhibitor of the hydroxynaphtalene reductase.

Results from flow cytometry experiments confirmed previous work which suggested that the laminin receptors were located on the ornamentations of the conidial wall. Scanning or transmission electron microscopy, showed that labelling was associated mainly with protrusions of the cell wall [21,22]. The marked decrease in laminin binding receptors to the surface of conidia of mutant isolates compared to reference strains, together with the smooth-walled appearance of these conidia, strengthens our previous conclusions. Previous work [10] also suggested the presence of at least two distinct adherence systems on the conidial surface in *A. fumigatus*: 1) the recognition of fibronectin from its tripeptide sequence Arg-Gly-Asp by two fungal polypeptides of 23 and 30 kDa, and 2) the binding of laminin and fibrinogen by a 72-kDa sialic acid-specific lectin located on the ornamentations of the conidial wall [23]. Our current results also support this hypothesis, showing a slight increase in the fibronectin binding capacity of mutant isolates compared with reference strains, together with a marked decrease in the binding of laminin to the conidial surface.

The physical properties of the surface of the conidia were also investigated, as they may contribute to host tissue adherence by bringing interacting surfaces closer and mediating their dehydration. We showed that blockage of the melanin biosynthesis pathway resulted in a marked decrease in the electronegative charge of the conidia, a charge which may be due to ionization of free amine and carboxylic acid groups of some surface proteins. A marked decrease in CSH was also observed for conidia of mutant isolates when compared to reference strains, which was consistent with the increased wettability of the colonies. This result suggests that blockage of the melanin pathway also led to the lack of some hydrophobic components on the conidial surface. The defect in melanin in *A. fumigatus *mutant isolates could also contribute to the marked loss of adherence properties of their conidia [24], as melanins are hydrophobic molecules and have a negative charge. Youngchim *et al*. [25] localised melanin in the electron dense outer layer of the cell wall which surrounds the conidia by TEM examination of *A. fumigatus *conidia before and after treatment with enzymes and hot acid. Nevertheless, the precise physico-chemical nature of melanin is not well defined and relationships between melanin and other components of the conidial wall, particularly polysaccharides, remain to be clarified [25,26].

Among the components of the conidial wall are small proteins called hydrophobins which have been described in a large variety of filamentous fungi including *A. fumigatus *[27]. Hydrophobins share some common properties. These moderately hydrophobic proteins are secreted into the environment by the fungus and they remain in a soluble form when the fungus is cultivated in a liquid medium. However, at an air-liquid interface (e.g. when the fungus is grown on a solid medium), they assemble in about 10-nm thick rodlets organised in bundles or fascicles on the conidial surface, forming a hydrophobic rodlet layer which may be visualised by AFM.AFM examination of the conidial surface showed that this rodlet layer was lacking in mutant isolates whereas typical rodlets were seen on conidia of the tested reference strain. Immunofluorescence or flow cytometry using specific anti-hydrophobin antibodies should be performed to determine whether or not hydrophobins are totally lacking at the conidial surface or simply not organised into a rodlet layer.

Conidia of *A. fumigatus *may germinate on contact with water. Previous studies showed major changes in the ultrastructure of the conidial wall during the first stage (swelling) of germination. In addition to a marked increase in cell size and the vacuolisation of the cytoplasm, TEM examination of swollen conidia showed changes in the cell wall which became thinner, probably due to the progressive detachment of the outermost cell wall layer [28]. Conidia of mutant isolates and of reference strains were also examined by SEM and AFM using laminin-coated glass coverslips applied to the centre of sporulating cultures. These experiments confirmed the smooth surface of the conidia of mutant isolates and showed the lack of rodlets at their surface. However, this study was conducted on clinical or environmental isolates with defective DHN-melanin pathways and no isogenic wild-type isolates were available as controls, so other mutations, besides those identified in the melanin pathway may have been responsible for phenotypic changes other than colony colour. Nevertheless, the role of melanin in the organisation of the conidial wall was established, because cultivation of reference strains in a medium containing DHN-inhibitors including pyroquilon led to smooth-walled conidia devoid of the outermost electron-dense layer.

## Conclusion

These results demonstrated that, as suggested by Franzen *et al*. for *Fonsecaea pedrosoi *[29], melanin is required for correct assembly of the different layers of the conidial wall in *A. fumigatus *and, therefore, for the expression of adhesins and other virulence factors at the conidial surface. Due to the complete lack of laminin binding at the surface of their conidia, these pigmentless isolates may be valuable tools in the characterisation of fungal receptors. Comparative studies of the proteins of these isolates and of reference strains are now being undertaken using 2D-electrophoresis.

## Methods

### Fungal strains

Unless otherwise specified, all experiments were conducted on three *Aspergillus fumigatus *isolates from the IHEM Culture Collection (Table 1) producing white (IHEM 2508, IHEM 9860) or brown (IHEM 15998) powdery colonies (Figure 2). Properties of these isolates were compared to those of the reference strain IHEM 18963 (Af293) previously used for genome sequencing of *A. fumigatus*. Likewise, strain CBS 113.26 previously used in our laboratory for studies on adherence mechanisms in *A. fumigatus *[9,21,30] was also included in these experiments. Both reference strains produced typical, dark blue-green powdery colonies.

### Media, growth conditions and preparation of conidial suspensions

Isolates were maintained by weekly passages on yeast extract-peptone-dextrose-agar (YPDA) plates containing in g/L: yeast extract, 5; peptone, 10; glucose, 20; and agar, 20. For some experiments, the organisms were also cultivated on Czapek agar (FeSO_4_, 7 H_2_O, 0.01 g; saccharose, 30 g; MgSO_4_, 0.5 g; KCl, 0.5 g; K_2_HPO_4_, 1 g; NaNO_3_, 3 g; agar, 20 g). Unless otherwise specified, all culture media were supplemented with chloramphenicol 0.5% and cultures were incubated at 37°C for 5 days. Conidia were harvested from 5-day-old cultures on YPDA plates, by scrapping off the mycelium in sterile distilled water, followed by filtration through 28-μm-pore-size nylon filters to eliminate pieces of agar, hyphal fragments and conidial heads. Cells were then pelleted by centrifugation (5 min at 1500 *g*), washed in sterile distilled water and finally counted using a haemocytometer.

### Effect of DHN-melanin inhibitors

Tricyclazole, pyroquilon and fenoxanil (Sigma-Aldrich) were diluted in ethanol and added to Czapek agar, at a final concentration of 20 μg/mL, according to the method of Cunha *et al*. [24]. Fungal suspensions were prepared as previously described from 5-day-old cultures. After 90 minutes decantation, 50 μL of the supernatant were applied to the surface of the agar plates. Cultures were incubated for 3 days at 37°C. Experiments were conducted in triplicate. Growth controls in Czapek agar without inhibitor and supplemented or not with ethanol, were included for each strain. Statistical analysis was applied, using the unpaired Student's *t-*test.

### DNA extraction and gene sequencing

The genomic DNA of the five strains was extracted using the DNeasy Plant Mini Kit (Qiagen Hilden, Germany) from mycelium previously ground in liquid nitrogen. Primers used for amplification of the *ALB1, AYG1*, *ARP1*, *ARP2*, *ABR1 *and *ABR2 *genes are listed in Table 6. They were designed with the WebPrimer program http://seq.yeastgenome.org/cgi-bin/web-primer from *A. fumigatus *B-5233 *ALB1*, *AYG1*,*ARP1*, *ARP2*, *ABR1 *and *ABR2 *gene sequences (Genbank accession numbers AF025541, AF116902, AFU95042, AF099736, AF116901, AF104823, respectively) and synthesized by Sigma-Aldrich. The PCR conditions, using Paq5000™ DNA Polymerase (Stratagene, La Jolla, CA, USA), were as follows: 2 min of denaturation at 95°C, followed by 30 cycles of 20 s at 95°C for denaturation, 20 s at a temperature between 50 and 58°C according to the melting temperature (Tm) of the primer for annealing and 30 s at 72°C for elongation, and by a final elongation step of 5 min at 72°C. The sequencing products, which were prepared in our laboratory, were analysed by Qiagen sequencing services (Hilden, Germany) on a ABI Prism 3700 DNA Analyzer (Applied Biosystems). In addition, the presence of conserved patterns and profiles was checked by scanning the aminoacid sequence with the InterProScan tool http://www.ebi.ac.uk/Tools/InterProScan/.

**Table 6:** Oligonucleotides used for gene sequencing

Gene name (gene product)	Genbank accesion no.	Primer	Nucleotide sequence (5'-3')	Nucleotide coordinates^1^
*AfALB1 *(polyketide synthase)	AF025541	ALB1-F	CAAACCACTCGCCATGGA	585-602
		ALB1-2R	TCGGAGCAGAAGCTGAGGATA	1459-1479
		ALB1-3F	AAACACTTCAAGGCTCCTGGA	1385-1405
		ALB1-4R	TTGATACGACCAGGCGTGAAT	2263-2283
		ALB1-5F	CGACGACTACCGTGAGATCAA	2194-2214
		ALB1-6R	CGCAGCAGAGAAGTTGTTGAT	3059-3079
		ALB1-7F	CGCAATGCTCATATTGCCT	2990-3008
		ALB1-8R	TGGATCGAGCAGATGTTGAA	3843-3863
		ALB1-9F	ACGCAGCAGTGTCAGATGG	3782-3800
		ALB1-10R	AAGAGCCACTCCATTGACCTT	4670-4690
		ALB1-11F	ACGGAAACACGGCGACATT	4596-4614
		ALB1-12R	CGATAATGTCATCCCCTTCA	5471-5490
		ALB1-13F	GCGACGCTACATACCAGACAT	5395-5415
		ALB1-14R	AGATCCATGCCAAGTGTCTCT	6267-6287
		ALB1-15F	ATTGACCCGAGCGACAACTT	6193-6212
		ALB1-16R	TTAGCCCATTTGCTGTCGTT	6958-6977
		ALB1-17F	ACTTCCTCGCCTTCATCGACT	6902-6922
		ALB1-R	TTCACCCCACTAGGAACTCAT	7249-7269
*AfAYG1 *(polyketide shortening)	AF116902	AYG1-F	ATGCCACGCTGGATCCTT	333-350
		AYG1-2R	ATGATCAGCACGATGGGGA	959-977
		AYG1-3F	CCCACATCCCCATTTACATC	905-924
		AYG1-R	TCAGTTCTTCGTCTTCGAAGG	1732-1752
*AfARP1 *(scytalone dehydratase)	AFU95042	ARP1-F	TCACACCACAATGGTCGAAA	321-340
		ARP1-R	CACATGAAATGGTACTTTTGC	955-975
*AfARP2 *(hydroxynaphtalene reductase)	AF099736	ARP2-F	ATGGTGAACACCTGCACCTAT	331-351
		ARP2-R	TCAGCATTCCAAATCCCCA	1134-1152
*AfABR1 *(vermelone dehydratase)	AF0116901	ABR1-F	ATGTTCCATTCCAGGGCTCT	248-267
		ABR1-1R	TCGTCGTCGTAGGCAAATG	730-748
		ABR1-2F	TGGTATCACTCGCACGAAAT	654-673
		ABR1-3R	TTGATGATGATCTCCACGACC	1520-1540
		ABR1-4F	AACGCTTCTAATGCGTCGAT	1461-1480
		ABR1-R	CTACGAGGCATTTGCGCAG	2323-2341
*AfABR2 *(oxydase)	AF104823	ABR2-F	ATACACGACAACAGGATGTGG	490-510
		ABR2-2R	TCAATTCCTCGGGGTCGT	1371-1388
		ABR2-3F	TTCCCACCAGATACAAGCTGA	1284-1304
		ABR2-4R	TTGCGGGTCGTGATCTTGA	2168-2286
		ABR2-5F	TAGCAACCTTGCTGCGTTG	2103-2121
		ABR2-R	GGGCAATCACATAGGAGTGA	2552-2571

^1 ^Nucleotide coordinates refer to the corresponding gene sequence in the Genbank database.

### Nucleotide sequence accession number

*ALB1, AYG1*, *ARP1*, *ARP2*, *ABR1 *and *ABR2 *gene sequences determined for strains or isolates CBS 113.26, IHEM 18963, IHEM 2508, IHEM 9860 and IHEM 15998 were deposited in the Genbank database and are available under accession numbers FJ406463 to FJ406498 (see Table 2).

### Scanning electron microscopy

Cultures grown through dialysis membranes, conidial suspensions, and conidia fixed on laminin-coated glass coverslips, were examined by SEM. Conidial suspensions were prepared as previously described. For the observation of conidial heads, cultures were grown on YPDA plates through sterile dialysis membranes. After 24 hours incubation, the membrane was removed from the agar plate and then cut into squares (0.5 cm × 0.5 cm) at the periphery of the colony. Round glass coverslips (12 mm diameter) were coated with 500 μL of a laminin solution (10 μg/mL final concentration) in phosphate buffered saline 0.15 M pH 7.2 (PBS) supplemented with 10 mM ethylene-diamine-tetraacetic acid (EDTA) to prevent polymerization of laminin. After 30 min incubation at 37°C under constant shaking, coverslips were washed in PBS. They were then directly applied to the surface of sporulating cultures, and finally washed to remove non adherent conidia.

All samples were fixed with a mix of 2% glutaraldehyde and 2% paraformaldehyde in phosphate buffer 0.1 M under vacuum for 24 hours. After washing, the cells were post-fixed with 2% osmium tetroxyde, then dehydrated by passage through ethanol solutions of increasing concentration (50 to 100%). Finally, ethanol was replaced with hexamethyldisilazane (HMDS) and samples were coated with carbon. Observations were made on a JSM 6301F scanning electron microscope (Jeol, Paris, France) operating at 3 kV and equipped with digital imaging.

### Flow cytometry analysis

Human plasma fibronectin and laminin from the murine Englebreth-Holm-Swarm sarcoma tumour (Sigma-Aldrich) were labelled with 5-fluorescein isothiocyanate (FITC; Sigma-Aldrich) by a procedure adapted from Clark and Shepard [31], as previously described [30]. Binding of laminin and fibronectin to the conidia was analysed by flow cytometry as described previously for *A. fumigatus *. In these assays, 10^7 ^conidia were incubated for 30 min at 37°C under constant shaking with 250 μL of FITC-conjugated protein solution (50 μg/mL final concentration). The cells were then washed, pelleted by centrifugation (3 min at 3500 *g*) and fixed with 1% formaldehyde in PBS. Experiments were performed in PBS (supplemented with 10 mM EDTA for laminin binding assays). Specificity of the binding was assessed by incubating the cells with the fluorescent laminin or fibronectin in the presence of a 10-fold excess of the same unlabeled protein. All experiments were carried out at least twice and included a negative control performed by incubating the cells with no ligand to ascertain the absence of autofluorescence. Cell surface fluorescence was quantified with a FacsCanto II flow cytometer (Becton-Dickinson). The significance of the difference between two fluorescence frequency distribution histograms (number of fungal cells versus relative fluorescence intensity expressed as arbitrary units on a logarithmic scale) was confirmed by statistical analysis using the Kolmogorov-Smirnoff two sample test. The data presented correspond to mean values of the cell surface fluorescence calculated, in all experiments, from the analysis of about 10,000 cells per sample.

### Microelectrophoresis

The net surface charge of the conidia was evaluated with a Zetasizer (Malvern Instruments, Worcestershire, United Kingdom) as described by Uyen *et al*. [32], by measuring the electrophoretic mobility of the cells in suspension in distilled water (10^7 ^conidia/mL). Data were collected from 5,000 cells, and the zeta potential was calculated for each strain using the Helmotz-Smoluchowski equation.

### Two-phase partitioning

The cell surface hydrophobicity (CSH) was first determined by two-phase partitioning as described by Kennedy *et al*. [33] with hexadecane as the hydrocarbon phase. Five hundred microliters of hexadecane were added to 2.5 mL of the conidial suspension (10^8^/mL) in phosphate buffered saline PBS. After vortexing the suspensions (2 min at 2200 vib/min), the tubes were incubated for 10 min at room temperature to allow the two phases to separate. The absorbance of the aqueous phase was then measured at 630 nm (Dynatech MRX revelation) and compared to that of a control consisting of a conidial suspension treated in the same conditions, but without hexadecane. CSH was also determined using a two-aqueous phase system adapted from Cree *et al*. [34] and consisting of a mix 1:1 of a 17.5% dextran 260,000 solution (900 μL) and a 14.26% polyethylene glycol (PEG) 3,350 solution (900 μL) in PBS. Two hundred microliters of the conidial suspension in PBS (10^7 ^conidia/mL) were added and the obtained suspensions were gently mixed. The tubes were then incubated for 1 hour at room temperature to allow the two phases to separate. Equal volumes (100 μL) of the upper phase rich in PEG (and therefore considered as hydrophobic) and of the lower phase rich in dextran (and therefore considered as hydrophilic) were then sampled and the absorbance of the two phases measured spectrophotometrically at 630 nm. CSH was expressed as the ratio between the absorbance of the upper phase and that of the lower phase.

### Transmission electron microscopy

The ultrastructure of the conidial wall was investigated by TEM using conidial suspensions obtained from 5-day-old cultures on YPDA as described above. Fixation, post-fixation, dehydratation and embedding in Epon were as previously described [22]. Thin sections contrasted with uranyle acetate and lead citrate were examined on a JEM-2010 transmission electron microscope (Jeol, Paris, France).

### Atomic force microscopy

Conidia were fixed on round glass coverslips coated with laminin, as previously described for MEB. The samples were washed in PBS buffer and then dried at room temperature before AFM analysis on a Thermomicroscopes Autoprobe CP Research (Veeco Instruments, Sunnyvale, CA, USA).

## Authors' contributions

All the authors participated in the study. JPB and FS designed the study protocol; MP was responsible for two-phase partitioning analysis and carried out the molecular analysis with PV; MP, GT, SG and RM were responsible for SEM, TEM and AFM analysis; MP and GR carried out the flow cytometry analysis; PS was responsible for microelectrophoresis. MP drafted the manuscript, JPB and DC critically reviewed the manuscript for its intellectual content and gave final approval of the version to be submitted. All authors read and approved the final manuscript.

## About the Authors

MP, GT, DC, FS and JPB are members of the ISHAM Working group on Chronic respiratory infections in cystic fibrosis.
